# “Rational” Management of Dichlorophenols Biodegradation by the Microalga *Scenedesmus obliquus*

**DOI:** 10.1371/journal.pone.0061682

**Published:** 2013-04-16

**Authors:** Aikaterini Papazi, Kiriakos Kotzabasis

**Affiliations:** Department of Biology, University of Crete, Voutes University Campus, Heraklion, Crete, Greece; Dowling College, United States of America

## Abstract

The microalga *Scenedesmus obliquus* exhibited the ability to biodegrade dichlorophenols (dcps) under specific autotrophic and mixotrophic conditions. According to their biodegradability, the dichlorophenols used can be separated into three distinct groups. Group I (2,4-dcp and 2,6 dcp – no *meta*-substitution) consisted of quite easily degraded dichlorophenols, since both chloride substituents are in less energetically demanding positions. Group II (2,3-dcp, 2,5-dcp and 3,4-dcp – one *meta*-chloride) was less susceptible to biodegradation, since one of the two substituents, the *meta* one, required higher energy for C-Cl-bond cleavage. Group III (3,5-dcp – two *meta*-chlorides) could not be biodegraded, since both chlorides possessed the most energy demanding positions. In general, when the dcp-toxicity exceeded a certain threshold, the microalga increased the energy offered for biodegradation and decreased the energy invested for biomass production. As a result, the biodegradation per cell volume of group II (higher toxicity) was higher, than group I (lower toxicity) and the biodegradation of dichlorophenols (higher toxicity) was higher than the corresponding monochlorophenols (lower toxicity). The participation of the photosynthetic apparatus and the respiratory mechanism of microalga to biodegrade the group I and the group II, highlighted different bioenergetic strategies for optimal management of the balance between dcp-toxicity, dcp-biodegradability and culture growth. Additionally, we took into consideration the possibility that the intermediates of each dcp-biodegradation pathway could influence differently the whole biodegradation procedures. For this reason, we tested all possible combinations of phenolic intermediates to check cometabolic interactions. The present contribution bring out the possibility of microalgae to operate as “smart” bioenergetic “machines”, that have the ability to continuously “calculate” the energy reserves and “use” the most energetically advantageous dcp-biodegradation strategy. We tried to manipulate the above fact, changing the energy reserves and as a result the chosen strategy, in order to take advantage of their abilities in detoxifying the environment.

## Introduction

Chlorinated phenolic compounds are found widely in the environment. The main sources are wood pulp bleaching, water chlorination, textile dyes, oil refineries, and chemical, agrochemical and pharmaceutical industries [1], [2], [3]. Chlorophenols have been shown to be toxic to terrestrial plants [4], [5], aquatic plants [6] and bacterial populations [2], [7]. Some of them are suspected to be endocrine disrupters and have adverse effects on humans and other organisms in the natural ecosystem at concentrations lower than the emission standard [8], [9], [10]. *In vitro* studies using isolated mitochondria and chloroplasts have shown that chlorophenols are potent uncouplers of oxidative and photosynthetic phosphorylation [11], [12], [13], thus inhibiting the formation of ATP. Therefore, they need to be removed from industrial effluents to ensure water pollution control.

There are several chemical and biological ways for degradation of chlorinated phenols. The most common chemical degradation processes of chlorinated phenols are the photo-dissociation [14], the photo-isomerization [15], the photo-substitution [16], the photo-rearrangement [17], the photo-oxidation [18], [19], [20] and the photo-reduction [21]. Despite being recalcitrant, chlorophenols can be degraded by fungi [1], [2], [3], [22] and bacteria [23], [24], [25]. Plants and actinomycetes can modify chlorophenols, often by making them more water-soluble [26], [27] and thus easier to degrade by microorganisms [28], [29], [30].

Considerable progress has been made in the isolation and investigation of bacterial strains, which degrade chlorophenols [31], [32], [33], [34], [35], [36], [37]. The pathways of their degradation are intensely investigated. Two routes of chlorophenol degradation are known: *via* the formation of chlorocatechols or hydroxyhydroquinol. In the first case, ring cleavage leads to the dechlorination of the aliphatic intermediates [38], [39]; in the latter, the initial substrate and its metabolites are dechlorinated before the ring cleavage [31], [32], [33], [40], [41], [42]. Nakagawa et al. (2006) [22] showed that the fungus *Mortierella* sp uses two different degradation pathways. In the first pathway *ortho*-oxidation resulted into two different dichloroguaiacols, while in the second pathway dechlorination was taken place before the ring cleavage. Mars et al. (1997) [43] have studied the microbial degradation of chloroaromatics and the use of *meta*-cleavage pathway for the mineralization of chlorinated compounds. They demonstrated the degradation studies with *Pseudomonas putida* GJ31 on toluene and chlorobenzene.

Nevertheless, since bacteria are heterotrophs and need organic nutrients to grow and degrade pollutants, their addition to the polluted matter is inevitable. Due to above, the application of a bacterial method for the practical remediation of pollutants at low concentrations is difficult. On the other hand, since microalgae are ubiquitous and can grow also autotrophically, they represent a practical way for the remediation of various pollutants [44]. Recently we showed that the biodegradation of phenolic compounds by microalgae seems to be not a simple feature of a particular organism, as it was thought to be before, but mostly a light dependent bioenergetic process [45]. That allows the utilization of cheap and abundant sunlight energy for the biodegradation procedure. Additionally microalgae can further used for producing alternative energy sources, since they can in parallel produce hydrogen [46], or they can used for biodiesel production [47] and antioxidants [48]. Despite the obvious advantage of the use of microalgae for pollutant removal, only a few studies have shown that microalgae are able to biodegrade aromatic compounds [45], [46], [49], [50], [51], [52], [53], [54], [55].

The present contribution is a continuation of our previews publications [45], [46], [50], [51] concerning the biodegradation of several phenolic compounds, where we showed that biodegradation is a photo- and carbon-source-regulated procedure. In the present work, phenols of higher toxicity (dichlorophenols) were tested for their biodegradability under autotrophic, heterotrophic and mixotrophic conditions. The results were used to investigate the correlation between the biodegradability and the toxicity of the phenolic compounds, referring to the comparison of the bioenergetic strategy of microalgae to biodegrade monochlorophenols (cps) (lower toxicity) and dichlorophenols (dcps) (higher toxicity). Finally, we tested all possible combinations of phenolic intermediates from the dcp-biodegradation pathway to check cometabolic interactions.

## Results

### The Substituent Pattern of dcps Adjust the Microalgal Growth

**Figure 1:**
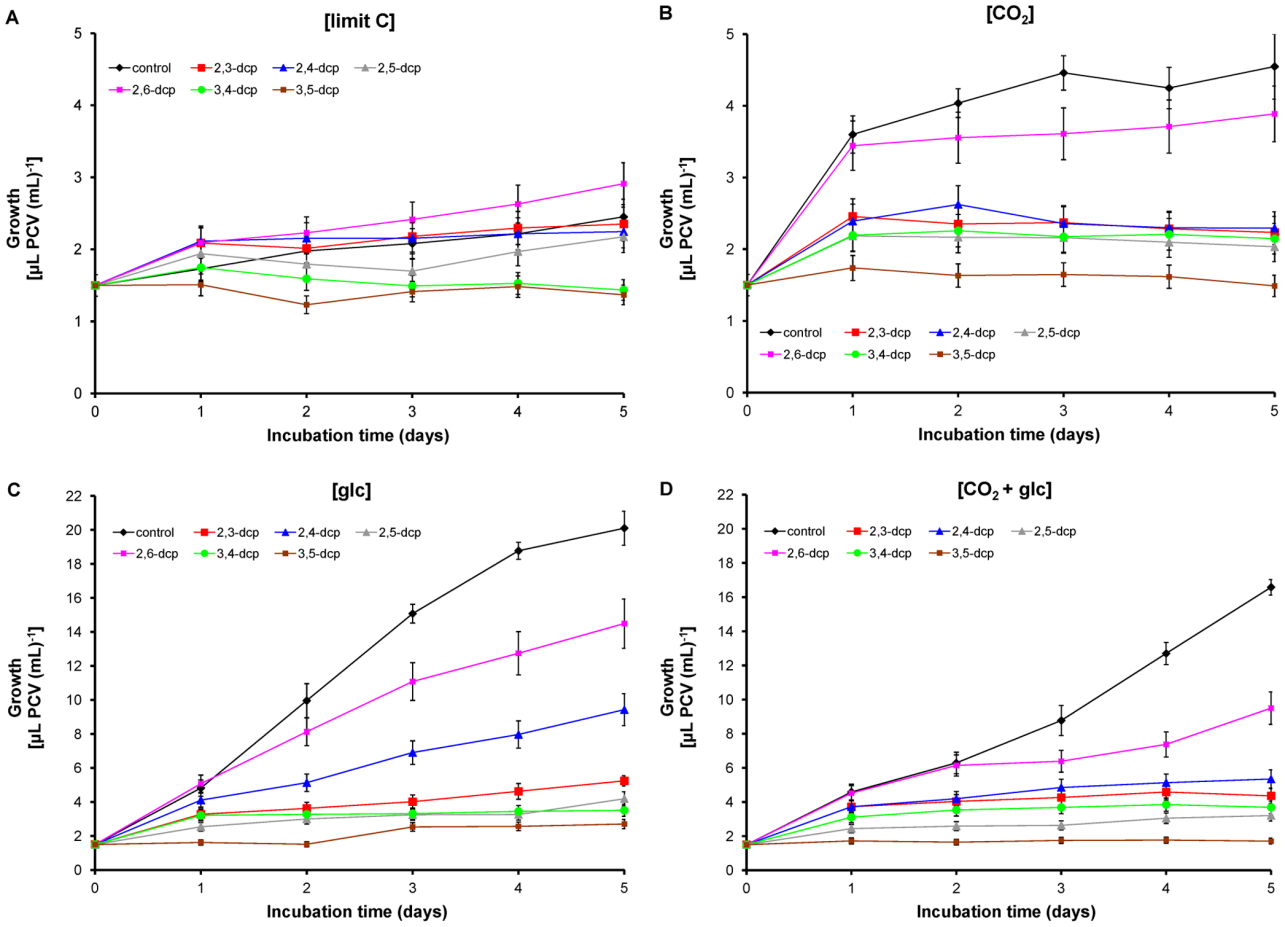
Growth curves of *Scenedesmus obliquus* cultures treated with dcps. (**A**) [limit C]-treatment, (**B**) [CO_2_]-treatment, (**C**) [glc]-treatment and (**D**) [CO_2_+glc]-treatment.

Cultures of *Scenedesmus obliquus* were supplied with different inorganic and organic carbon sources ([CO_2_]-, [glc]-, [CO_2_+glc]- and [limit C]-treatment – s. Materials and Methods). All possible combinations of the chlorine substituents of dcps, 2,3-, 2,4-, 2,5-, 2,6-, 3,4- and 3,5-dcp, were tested for their influence on the algal growth (Figure 1). Independently of the applied dcp, the best biomass increase was achieved in cultures incubated with glucose ([glc]-treatment), followed by [CO_2_+glc]- and [CO_2_]-treatment, while the least growth was obtained with [limit C]-treatment (Figure 1). The algal growth was inhibited, to a different extent, by dcps. This observation was verified in all the tested carbon sources. Among dcps, 2,6-dcp and 2,4-dcp achieved the least algal growth inhibition, while the inhibition observed in all the other tested dcps was much higher.

### Changes in the Molecular Structure and Function of the Photosynthetic Apparatus in Dependence of the Added dcps and the Exogenously Supplied Carbon Source

The changes induced in the molecular structure and function of the photosynthetic apparatus by dcps were estimated using fluorescence induction measurements and the JIP-test in the first 5 h (Table 1). The mother culture was started with F_v_/F_m_ = 0.701, while after the first 5 h the control values were quite lower in the applied carbon sources, possibly because of the conditions established in the hermitically closed bottles of the experimental procedures.

**Table 1:** Photosynthetic parameters of *Scenedesmus obliquus* in the four experimental treatments with different carbon source in the presence of dcps for 5 h. F_v_/F_m_: photosynthetic efficiency (STDEV: 0.001–0.012), ABS/RC: antenna size (STDEV: 0.5–1.5), DIo/RC: dissipation energy per reaction center (STDEV: 0.5–1.1), RC/CS_o_: density of active reactions centers (STDEV: 1.5–5).

[limit C]					[CO_2_]				
	Fv/Fm	ABS/RC	DIo/RC	RC/CSo		Fv/Fm	ABS/RC	DIo/RC	RC/CSo
**control**	0.654	2.269	0.815	24.679	**control**	0.669	1.976	0.679	28.843
**2,3-dcp**	0.636	2.575	1.004	17.863	**2,3-dcp**	0.671	2.876	1.048	17.732
**2,4-dcp**	0.648	3.073	1.147	17.250	**2,4-dcp**	0.638	2.756	1.042	17.414
**2,5-dcp**	0.579	3.057	1.343	15.373	**2,5-dcp**	0.594	3.047	1.297	14.112
**2,6-dcp**	0.609	1.862	0.742	28.464	**2,6-dcp**	0.560	3.165	1.481	16.114
**3,4-dcp**	0.495	3.757	2.064	16.239	**3,4-dcp**	0.605	2.633	1.061	19.751
**3,5-dcp**	0.504	1.991	1.039	30.135	**3,5-dcp**	0.510	3.403	1.767	15.866
**[glc]**					**[CO_2_+glc]**				
	**Fv/Fm**	**ABS/RC**	**DIo/RC**	**RC/CSo**		**Fv/Fm**	**ABS/RC**	**DIo/RC**	**RC/CSo**
**control**	0.664	1.932	0.675	26.394	**control**	0.652	1.616	0.584	34.650
**2,3-dcp**	0.618	3.173	1.327	14.498	**2,3-dcp**	0.664	2.148	0.773	20.946
**2,4-dcp**	0.589	2.333	1.000	20.574	**2,4-dcp**	0.640	2.848	1.048	16.153
**2,5-dcp**	0.548	3.115	1.468	15.732	**2,5-dcp**	0.564	2.160	0.984	21.297
**2,6-dcp**	0.595	2.008	0.866	24.898	**2,6-dcp**	0.609	2.376	0.947	23.152
**3,4-dcp**	0.505	3.157	1.657	16.790	**3,4-dcp**	0.590	2.674	1.150	16.084
**3,5-dcp**	0.451	3.302	1.778	14.840	**3,5-dcp**	0.486	2.797	1.464	20.020

According to the JIP-parameters, it was observed that if one of the two chlorine substituents was in the *meta*-position [the 3^rd^ or the 5^th^ position of the phenolic ring - (2,3-dcp, 2,5-dcp and 3,4-dcp)], then the microalga was led to the inactivation of the reaction centers (RC/CS_o_), increase of the functional antenna size (ABS/RC), enhancement of the dissipation energy (DI_o_/RC) and subsequently, decrease of the photosynthetic efficiency (F_v_/F_m_). These changes induced in the photosynthetic apparatus expressed the toxicity (stress effect), caused by the presence of one *meta* substituted dcp compared to control. If two chlorides possessed a *meta*-position (3,5-dcp), then the stress effects were more intense for the first 5 h (Table 1). The repetition of the same experimental procedure after 24 h, showed complete inactivation of photosystem II [32]. Finally, dcps without any substituent in the *meta*-position, like 2,4- and 2,6-dcps, resulted in lower or no toxicity in the majority of the tested treatments.

**Figure 2:**
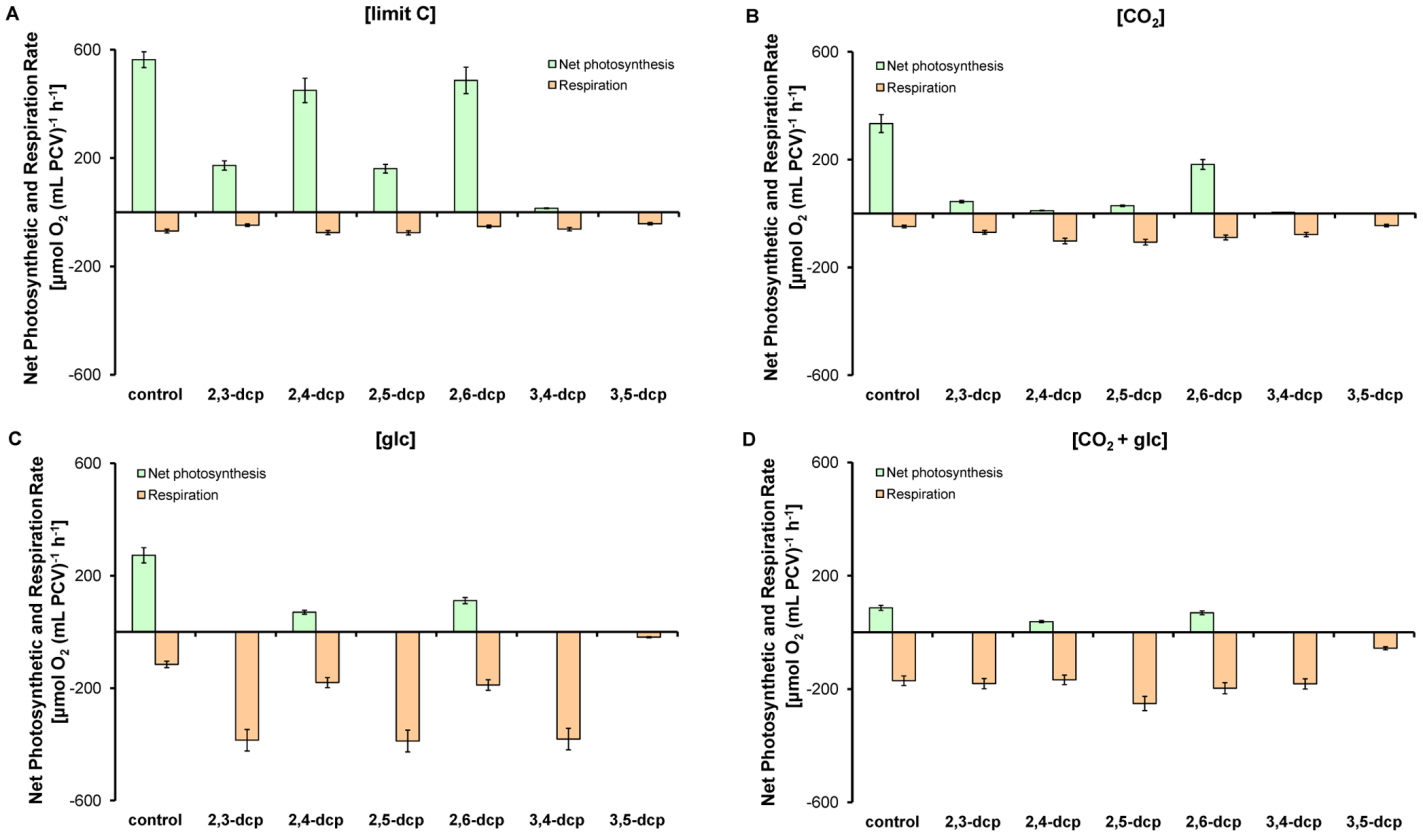
Polarographic determination of the maximal net photosynthetic and respiratory rates of *Scenedesmus* cultures treated with dcps. (**A**) [limit C]-treatment, (**B**) [CO_2_]-treatment, (**C**) [glc]-treatment and (**D**) [CO_2_+glc]-treatment. The measurements were taken place the fifth incubation day. The values that are not presented in the diagrams are so low that are fitted in with the x-axes.

The above results on the dcps toxicity were confirmed by the measurements of the maximal photosynthetic rate (Figure 2). Maximal values of net photosynthetic activity were obtained with dcps without any substituents in the *meta*-position, like the 2,4- and 2,6-derivatives. On the contrary, in all the other cases the net photosynthetic activity was rather low (Figures 2A and B) or zero (Figures 2C and D). This is an expected result since the JIP-test parameters revealed the extensive inhibition of photosystem II. Finally, it is important to point out that mainly in [glc]-treatment the respiration rates were extremely high when one of the two chlorine substituents possessed the *meta*-position of the phenolic ring (2,3-, 2,5- and 3,4-dcps; Figure 2).

Conclusively, there is a gradient in the toxicity (expressed as growth inhibition or changes in the molecular structure and function of the photosynthetic apparatus) of the six dichlorophenols that became obvious in all the experimental procedures. Its toxicity sequence being: no *meta*-substitute (2,6-dcp and 2,4-dcp)<one *meta*-substitute (2,3-dcp, 2,5-dcp and 3,4-dcp)<two *meta*-substitutes (3,5-dcp).

### Photoregulated dcp-biodegradation

**Figure 3:**
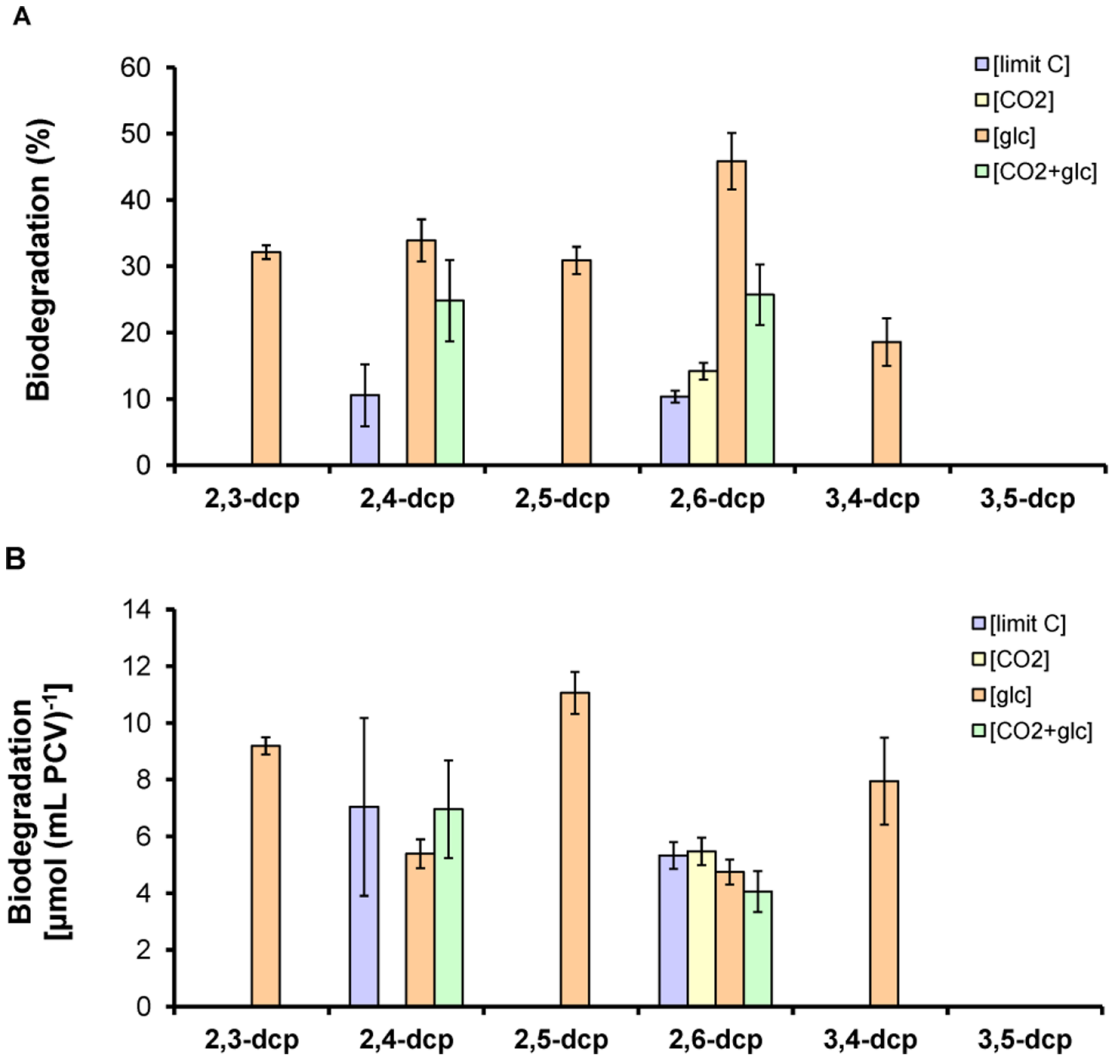
Removal of dcps under different carbon sources. The diagram (**A**) represents the biodegradation per culture, and the diagram (**B**) the biodegradation per packed cell volume under [limit C]-treatment, [CO_2_]-treatment, [glc]-treatment and [CO_2_+glc]-treatment after the fifth incubation day. The values that are not presented in the diagrams are so low that are fitted in with the x-axes.

**Figure 4:**
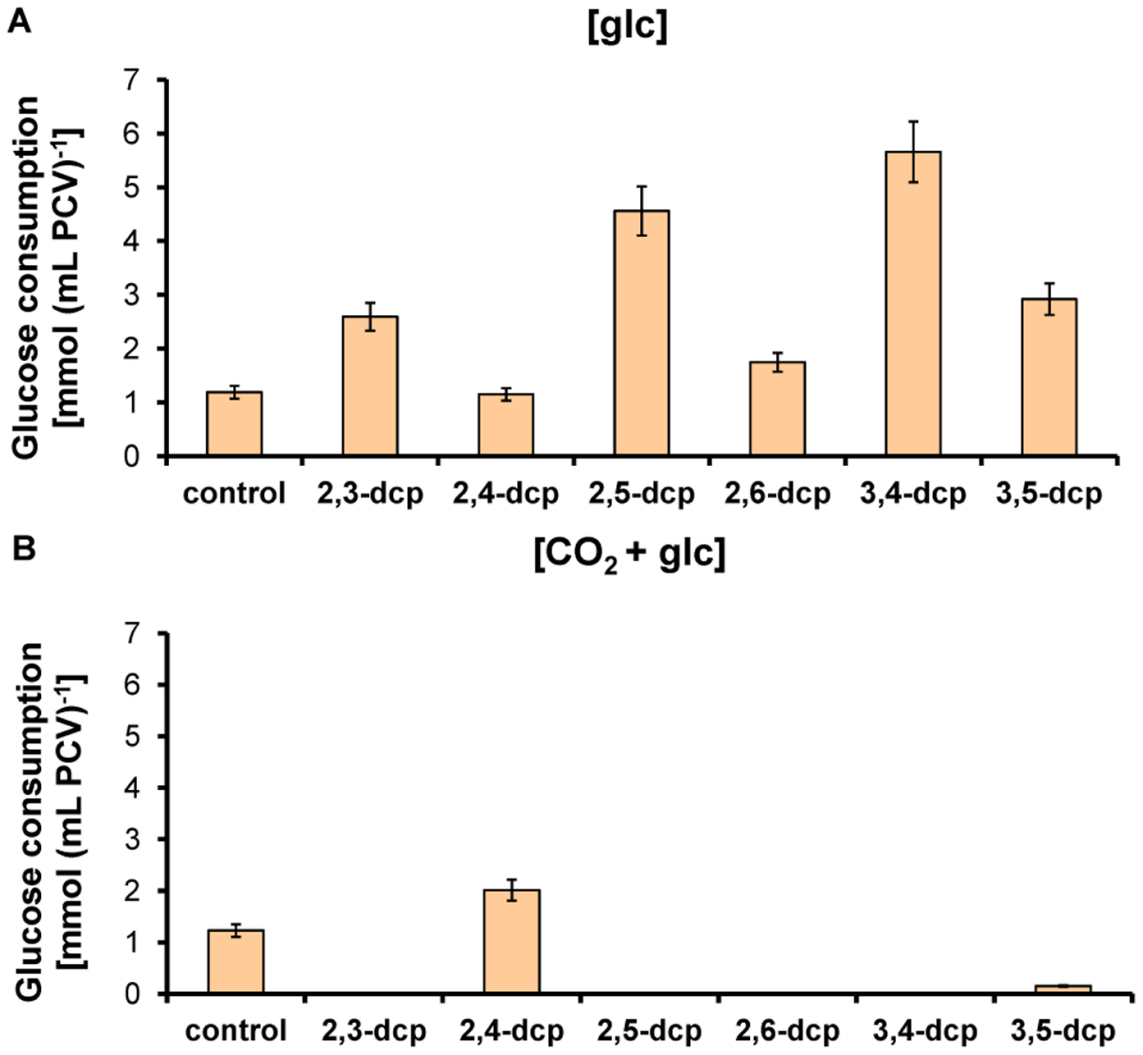
Glucose consumption of *Scenedesmus* cultures treated with different dcps. (**A**) [glc]-treatment and (**B**) [CO_2_+glc]-treatment. The values that are not presented in the diagrams are so low that are fitted in with the x-axes.

The removal of dcps after five days of incubation is illustrated in Figure 3. The first diagram represents the biodegradation per culture (50 mL) (Figure 3A) and the second the biodegradation per packed cell volume (Figure 3B). The maximum removal was observed with 2,3-, 2,5- and 3,4-dcps in mixotrophically grown cultures (glucose as exogenously supplied carbon source; [glc]-treatment). Measurements of glucose consumption of the cultures incubated with the different dcps, confirmed the participation of glucose to the biodegradation of one *meta*-substituted dcps (2,3-dcp, 2,5-dcp and 3,4-dcp) (Figure 4A). In all the other treatments, none of the three derivatives was degraded. In [CO_2_]- and [limit C]-treatment there is no glucose in the culture medium, while in the [CO_2_+glc]-treatment the glucose could not be consumed by the microalga (Figure 4B). Concerning the biodegradation of 2,4- and 2,6- dcp, the percentage of removal was similar at all carbon source treatments, except for 2,4-dcp which on [CO_2_]-treatment could not be removed (Figure 3). The last tested derivative (3,5-dcp) did not show any degradation in each of the four different treatments (Figure 3). A similar experiment was conducted in absolute darkness but the biodegradation rate was lower than 5% compared to the corresponding light values (data not shown). As a result, the biodegradation of dcps by *Scenedesmus obliquus* is considered to be mainly a light dependent process. This is in agreement with our previews contribution regarding the photoregulation of the biodegradation procedure of mono-halophenols [45].

### Comparative Study of the Biodegradation of dcps and the Corresponding Monochlorophenols – A Similar Strategy with an Unequal Energy Demand

**Figure 5:**
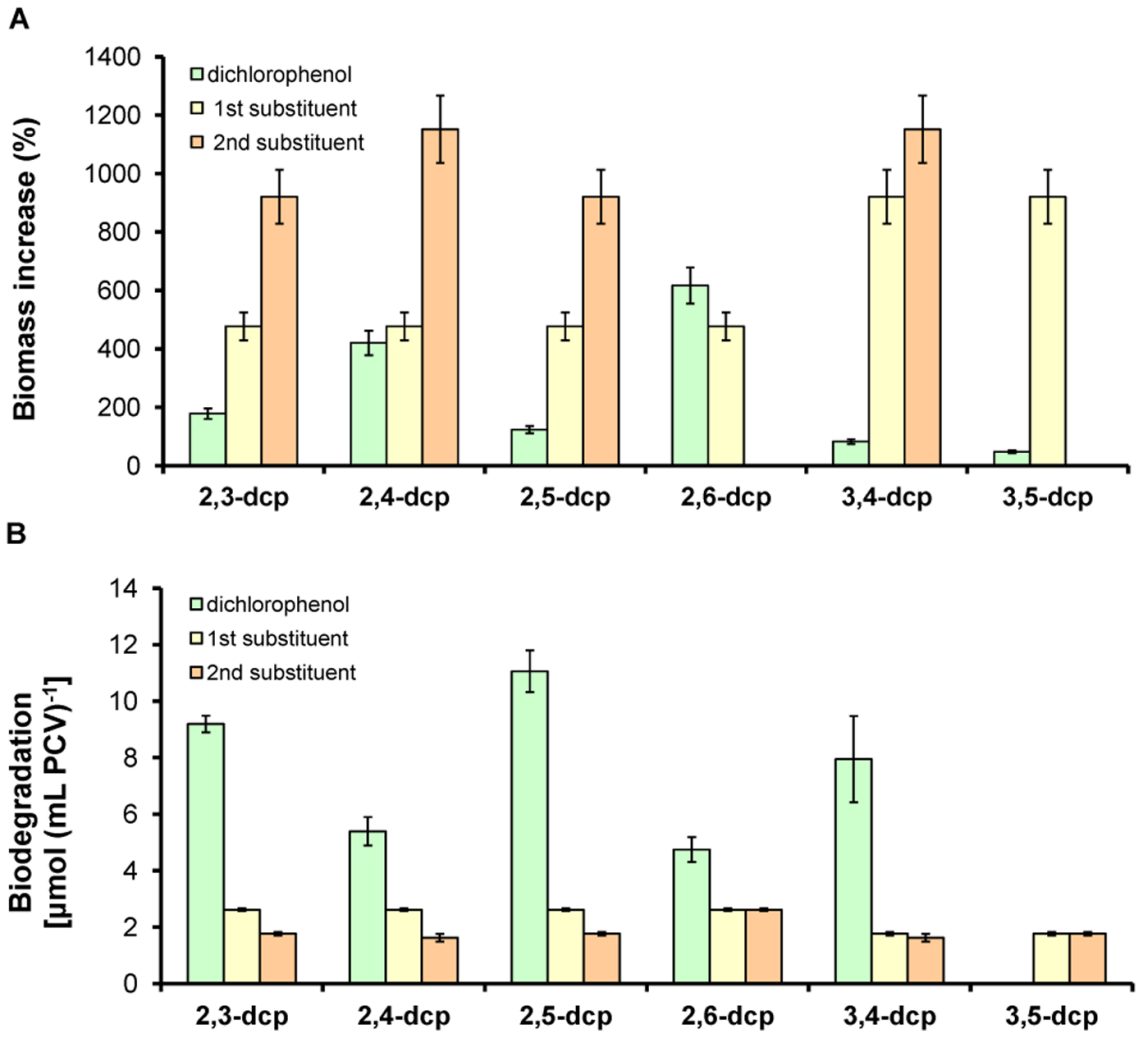
Comparative biodegradation strategy between cps and dcps in [glc]-treatment. (**A**) Biomass increase (change of the biomass in percentage between the fifth experimental day and the start of the experiment) of dcps versus the corresponding cps (those that a dcp could be divided into) at the same experimental conditions. [2,3-dcp was compared with 2-cp and 3-cp, 2,4-dcp with 2-cp and 4-cp, 2,5-dcp with 2-cp and 3-cp (5-cp is the structure of 3-cp), 2,6-dcp with 2-cp (6-cp is the structure of 2-cp), 3,4-dcp with 3-cp and 4-cp, while 3,5-dcp with 3-cp (5-cp is the structure of 3-cp)]. (**B**) Biodegradation per packed cell volume of dcps versus the corresponding cps (as explained above) at the same experimental conditions. The values that are not presented in the diagrams are so low that are fitted in with the x-axes.

The majority of the dcps could be degraded in [glc]-treatment, as indicated in Figure 3. Hence, [glc]-treatment was chosen in order to compare the biodegradation of dichlorophenols (dcps) and the corresponding mono-chlorophenols (cps) (those that could make up the dcps) exclusively under the above described experimental conditions (Figure 5).

Biomass production showed clearly that dcps are more toxic (more inhibition in biomass growth) for the microalga than the corresponding cps (Figure 5A). Despite the toxicity, all dcps, except the 3,5-derivative, yielded a significantly higher level of biodegradation per packed cell volume, compared to the corresponding cps (Figure 5B). Possibly the increased toxicity caused by the second chlorine substituent was the main reason that forced the microalga to activate survival mechanisms to detoxify its environment without investing to biomase increase. In case of lower toxicity (cps) the above mentioned mechanism took place in reverse order, since the microalga invested to biomass increase and maintained the biodegradability in low levels (Figure 5B).

### Cometabolism as a Bioenergetic Strategy of Combinational Biodegradation of Different Phenolic Compounds

The biodegradation procedure of dichlorophenols is a dynamic phenomenon that convert dcps to several intermediates (like the corresponding chlorophenols and phenol), which in turn can interact and influence positive or negative the dcps biodegradation. For this reason, we tested the biodegradability of 2,4-dcp in all the possible combinations of the other three possible phenolic derivatives (2-cp, 4-cp and phenol). The 2-cp and the 4-cp were chosen because they are the two mono-chlorophenols that 2,4-dcp consists of. Phenol was tested because after the split of the chloride substituents it could be an intermediate byproduct, before the final mineralization.

In the [limit C]-treatment (low energy reserves) the microalga biodegraded phenol (9.8%) and 2,4-dcp (10.5%) in low percentages, while 2-cp and 4-cp could not be degraded (Table 2). In combination treatments (two or more phenolic compounds), if 2,4-dcp did not exist in the tested phenolic combination then no biodegradability took place in the above mentioned low energy conditions. The only case of a parallel biodegradation was in the presence of 2,4-dcp with phenol or 4-cp (Table 2).

**Table 2:** Combinational biodegradation (per culture and per packed cell volume) of phenol, 2-cp, 4-cp and 2,4-dcp in [limit C]-, [CO_2_]- and [glc]-treatment.

[limit C]	Biodegradation per culture [%]				Biodegradation [µmol (mL PCV)^−1^]			
	phenol	2-cp	4-cp	2,4-dcp	phenol	2-cp	4-cp	2,4-dcp
**phenol**	9.8				9.2			
**2-cp**		0				0		
**4-cp**			0				0	
**2,4-dcp**				10.5				7.0
**phenol+2-cp**	0	0			0	0		
**phenol+4-cp**	0		0		0		0	
**phenol+2,4-dcp**	96.1			16.1	99.9			16.8
**2-cp+4-cp**		0	0			0	0	
**2-cp+2,4-dcp**		0		12.8		0		13.1
**4-cp+2,4-dcp**			14.1	21.1			12.9	19.3
**phenol+2-cp+4-cp**	0	0	0		0	0	0	
**phenol+2-cp+2,4-dcp**	0	0		14.0	0	0		15.0
**phenol+4-cp+2,4-dcp**	0		0	10.5	0		0	11.4
**2-cp+4-cp+2,4-dcp**		0	0	16.6		0	0	17.5
**phenol+2-cp+4-cp+2,4-dcp**	0	0	0	17.5	0	0	0	17.7
**[CO_2_]**	**Biodegradation per culture [%]**				**Biodegradation [µmol (mL PCV)^−1^]**			
	**phenol**	**2-cp**	**4-cp**	**2,4-dcp**	**phenol**	**2-cp**	**4-cp**	**2,4-dcp**
**phenol**	0				0			
**2-cp**		16				6.3		
**4-cp**			10				4.0	
**2,4-dcp**				0				0
**phenol+2-cp**	0	16			0	6.3		
**phenol+4-cp**	0		10		0		4.0	
**phenol+2,4-dcp**	11.3			25.8	15.6			35.4
**2-cp+4-cp**		0	0			0	0	
**2-cp+2,4-dcp**		0		0		0		0
**4-cp+2,4-dcp**			20.1	12.7			29.6	18.7
**phenol+2-cp+4-cp**	0	0	0		0	0	0	
**phenol+2-cp+2,4-dcp**	0	0		0	0	0		0
**phenol+4-cp+2,4-dcp**	11.0		21.6	10.7	16.9		33.3	16.4
**2-cp+4-cp+2,4-dcp**		0	12.9	9.0		0	17.8	12.4
**phenol+2-cp+4-cp+2,4-dcp**	13.8	0	17.5	10.2	21.7	0	27.5	16.1
**[glc]**	**Biodegradation per culture [%]**				**Biodegradation [µmol (mL PCV)^−1^]**			
	**phenol**	**2-cp**	**4-cp**	**2,4-dcp**	**phenol**	**2-cp**	**4-cp**	**2,4-dcp**
**phenol**	0				0			
**2-cp**		15.4				2.6		
**4-cp**			20.9				1.6	
**2,4-dcp**				33.9				5.4
**phenol+2-cp**	0	15.4			0	2.6		
**phenol+4-cp**	0		20.9		0		1.6	
**phenol+2,4-dcp**	0			24.1	0			12.8
**2-cp+4-cp**		8.5	12.3			1.0	1.5	
**2-cp+2,4-dcp**		21.5		27.5		3.7		12.9
**4-cp+2,4-dcp**			19.9	45.2			12.1	27.5
**phenol+2-cp+4-cp**	0	8.5	12.3		0	1.0	1.6	
**phenol+2-cp+2,4-dcp**	0	25.1		54.5	0	4.3		30.0
**phenol+4-cp+2,4-dcp**	0		10.5	30.3	0		5.8	16.8
**2-cp+4-cp+2,4-dcp**		7.5	7.4	18.7		4.9	4.9	12.4
**phenol+2-cp+4-cp+2,4-dcp**	0	5.9	7.1	12.9	0	4.3	5.2	9.4

In [CO_2_]-treatment (medium energy reserves) the microalga biodegraded 2-cp (16%) and 4-cp (10%), while phenol and 2,4-dcp could not be biodegraded (Table 2). In a higher toxicity level (in the presence of two or more phenolic compounds) the microalga biodegraded the most toxicant (2,4-dcp) phenolic compound, only in the presence of phenol or 4-cp or both of them in the culture medium.

In [glc]-treatment (high energy reserves) the microalga biodegraded 2-cp (15.4%), 4-cp (20.9%) and 2,4-cp (33.9%), but not phenol (Table 2). In a higher toxicity level (in the presence of two or more phenolic compounds) the microalga biodegraded all the tested phenolic compounds except phenol (Table 2). More energy reserves permitted more phenolic compounds biodegradability, as it was expected.

## Discussion

The experimental procedure shows clearly that dcps, according to their biodegradability, can be divided into three distinct groups. The separation refers to the absence or the presence of one, or two *meta*-substituted chlorides in the phenolic ring. The *meta*-chloride seems to be the regulated parameter for dcps biodegradability, since the *meta*-position is more energy demanding than the *ortho* and *para* ones [45]. The above mentioned in combination with the different exogenous supplied carbon-energy sources ([CO_2_]-, [glc]-, [CO_2_+glc]- and [limit C]-treatment) led to different biodegradation levels (Figure 3).

The first group (I) is represented by 2,4- and 2,6-dcp, since there is no *meta*-substituted chloride. Both of them were degraded quite easily under all applied experimental conditions ([limit C]-, [CO_2_]-, [glc]- and [CO_2_+glc]-treatment). Gomes and Ribeiro da Silva (2003) [56] calculated the thermodynamic properties of dcps. From the compilation of bond dissociation energies (BDE), it can be deduced that *ortho*-substitution always destabilizes the O-H bond. This is due to the repulsive steric interaction that is relieved upon O-H bond cleavage. The change in the bond dissociation energy (ΔBDEs) of 2,n-dcps (n = 3,4,5 or 6 position in the phenolic ring) compared to phenol, is smaller when the second chlorine atom enters positions 4 or 6 (as in our group) than 3 or 5. This is due to the fact that substituents at *para*- and *ortho*-positions can engage in resonance effects that are significantly diminished if the substituents go to the *meta*-position [56].

The above derivatives belong to the same group regarding the absence of a *meta*-chloride, but they have differences in the toxicity. 2,4-dcp exhibits higher growth inhibition compared to 2,6-dcp (Figure 1) and the photosynthetic apparatus of 2,4-treated cells is more stressed than the corresponding of 2,6-treated cultures (Table 1, Figure 2). The main difference between 2,4- and 2,6-dcp is that the second chloride possesses the *para*- and the *ortho*-position correspondingly. The above fact causes bioenergetic changes that resulted in biodegradability (Figure 3). Tomasi et al. (1995) [57] indicated that whatever the degree of halogenation, all the halophenols seem to be metabolized by the same enzyme that catalyzes the *para-*hydroxylation of the phenol ring, regardless of whether that position was originally replaced by a chlorine in the starting product. It is obvious that in 2,4-dcp the *para*-position has a chlorine atom so the energy demanded for the biodegradation is expected to be higher compared to 2,6-dcp, that has no chlorine in the *para-*position. This information agrees with Steiert et al. (1987) [58] since they indicated that toxicity increased with the degree of chlorination, but phenols chlorinated in positions 2 and 6 were least toxic.

The second group (II) is represented by 2,3-, 2,5- and 3,4-dcps, in which one of the two chlorine substituents is in *meta*-position (more energy demanded position for fission) [45]. Therefore, group II is more difficult to degrade compared to the above group I. As explained above, 2,n–dcps (n = 3,4,5 or 6 position in the phenolic ring) biodegraded easier than all the other dcps, because of the one, at least, *ortho*-substitution. Also, Gomes and Roberto da Silva (2003) [56] indicated that when chlorine atoms are placed in adjacent positions, they do not interact with each other, since the energetic difference between the two conformations of 2-cp and 2,3-dcp is almost the same. The above mentioned explain the biodegradation trend (2,3-dcp>2,5-dcp>3,4-dcp) among the three dcps of group II that appeared in Figure 3. It is worth mentioning that dcps of group II can only biodegraded in the presence of glucose (Figure 3, Figure 4A). This observation is in agreement with Petroutsos et al. (2008) [53], who indicated that these dcps followed glucosidation in order to biodegrade.

In [CO_2_+ glc]-treatment there is no biodegradation in group II, nevertheless glucose existed in the culture medium. The presence of CO_2_ in the culture medium favors photosynthesis while the presence of glucose favors respiration (Figure 2). This controversial situation does not permit the consumption of glucose (Figure 4B) so as the realization of the biodegradation procedure.

The third group (III) is represented by the most toxic 3,5-dcp, due to the presence of both *meta*-substituents in the phenolic ring. The extreme toxicity of the 3,5-dcp can explain the inability of the microalgae to degrade this phenolic compound under the applied experimental conditions.

Boyd and Shelton (1984) [59] indicated that dcps were bioconverted to cps. Especially, 2,6-dcp is converted to 2-cp, 2,4-dcp to 4-cp, 2,3-dcp and 2,5-dcp to 3-cp. Uchida and Okuwaki (2003) [60] calculated the bond dissociation energies of cps in several temperatures. In our situation ΔΗ(*ortho*) = 25.2 kJ/mol, ΔΗ(*para*) = 28.8 kJ/mol and ΔΗ(*meta*) = 36.1 kJ/mol. The above mentioned are in absolute agreement with our indications regarding to the difficulty in biodegradability of dcps.

**Figure 6:**
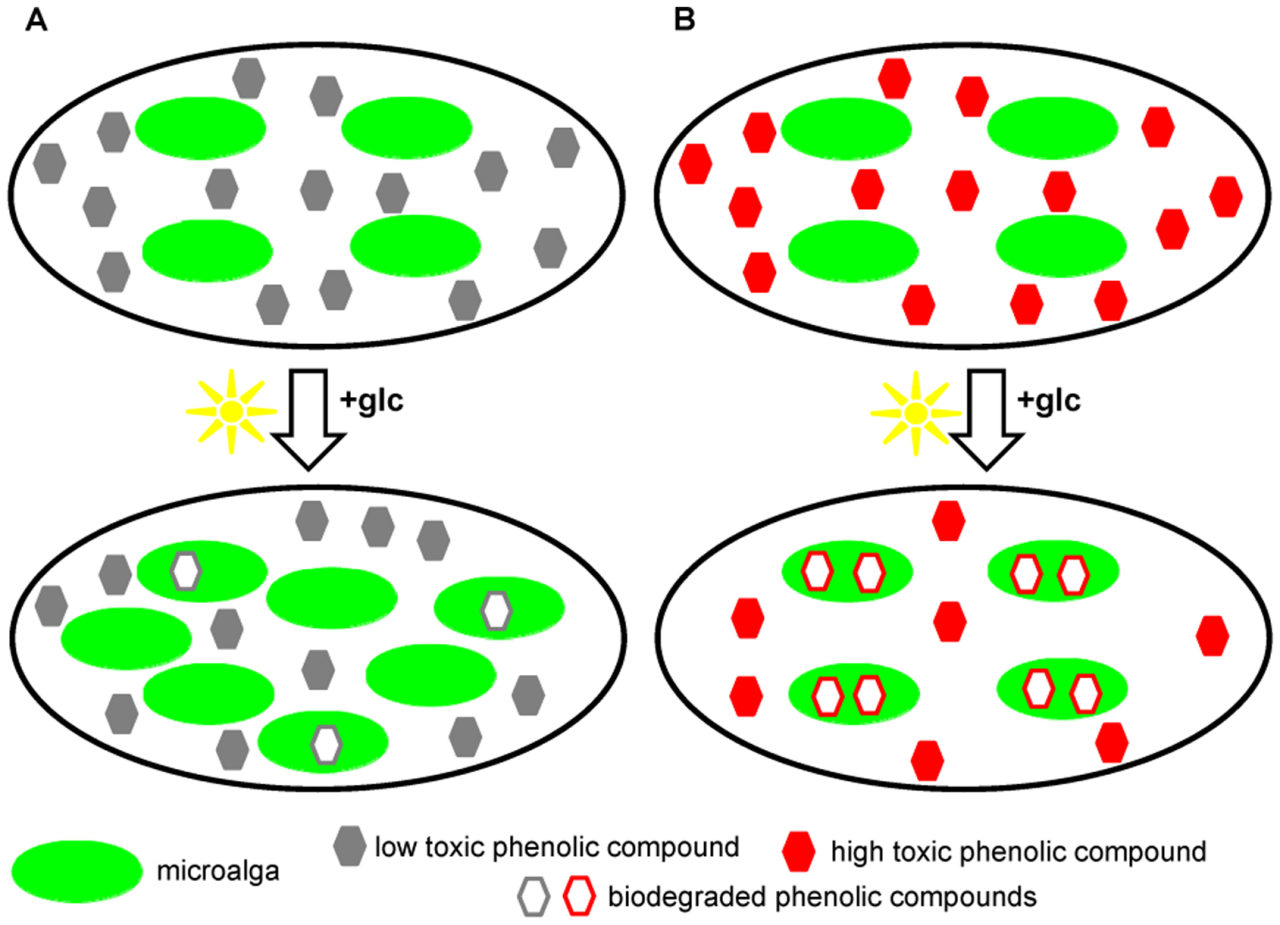
A simplified model for optimal management among dcp-toxicity, dcp-biodegradability and culture growth. The main purpose for both strategies (lower or higher toxicity) is the detoxification of the algal medium environment. Algal cells seem to be tolerant in the lower toxicity molecules (like cps or dcps of group I) and invest their energy mainly to algal growth rather than biodegradability. As a result, after an incubation time, the culture has more cells than the starting time point and each cell has to “face” lower quantity of toxic molecules. Therefore the cell tolerance increases without the need to invest additional energy for biodegradation, since the increasing growth is the strategy for facing the toxicity (indirect detoxification). On the other hand under higher toxicity (like dcps of group II), the microalga has to consume more energy to directly detoxify its culture environment (biodegradation) rather than “investing” on growth (direct detoxification).

**Figure 7:**
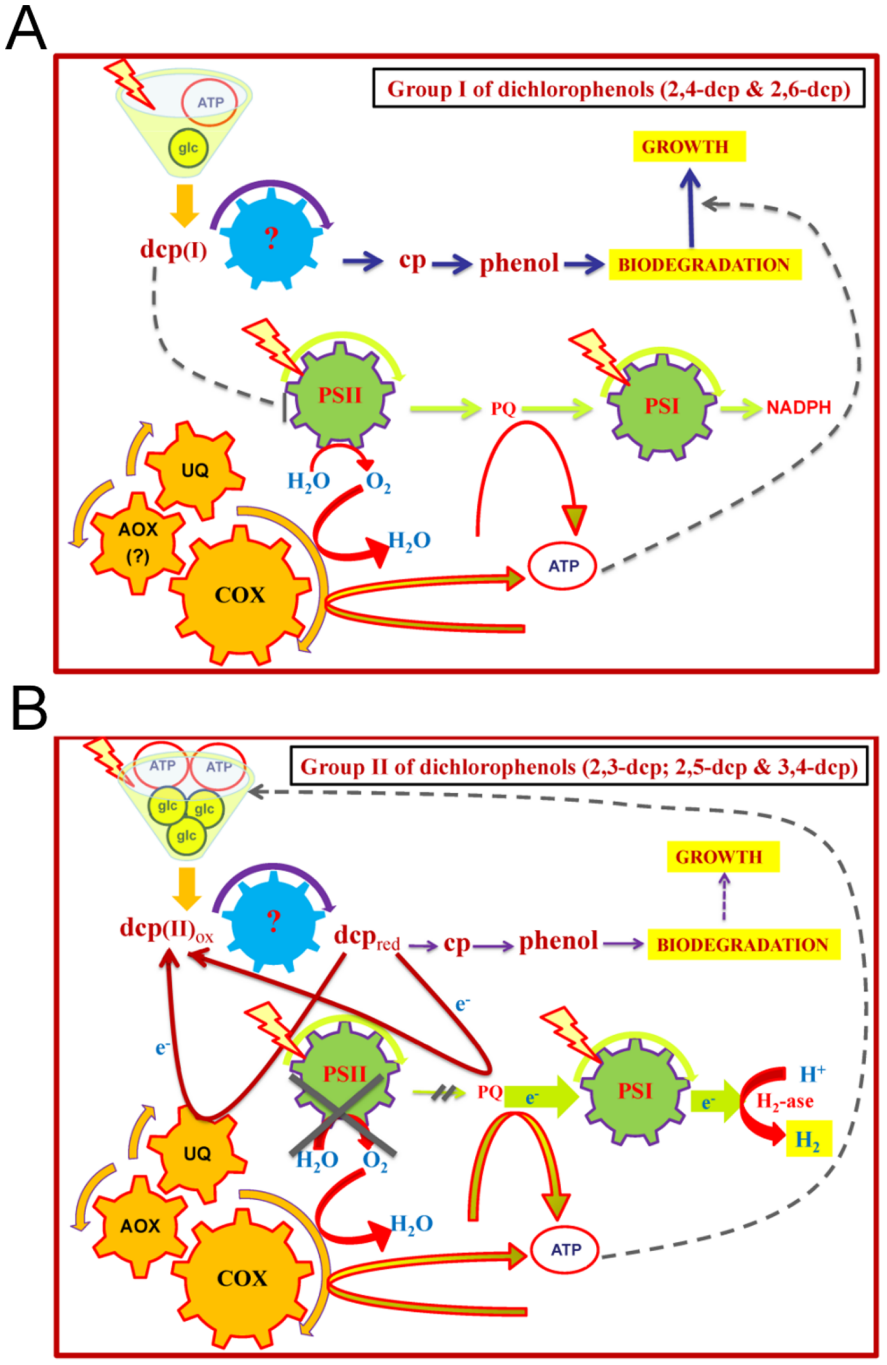
Group I and group II comparative bioenergetic models. (**A**) Group I consists of dcps with lower toxicity (2,4-dcp and 2,6-dcp). They reduce the photosynthetic activity (Figure 2) and the produced energy from photosynthesis and respiration is invested mainly to growth rather than to biodegradability (Figure 6). (**B**) Group II consists of dcps with higher toxicity (2,3-dcp, 2,5-dcp and 3,4-dcp). The biodegradation of group II seems to be more complicated. Recently, we found that reduced dcps of group II, according to their redox potential, take place as electron donors to the photosynthetic electron flow, close to the plastoquinone pool (PQ). In parallel, they block the activity of photosystem II and the release of O_2_, leading to the establishment of oxygen-depleted conditions. Additionally, the first step of dcps-biodegradation is the dcps-reduction that supports a continuous circuit between oxidized and reduced dcps, which continuously promotes strong electron flow to PQ-pool, and in turn to ferredoxin (Fd). As a result, hydrogen production is induced strongly and continuously by the hydrogenase activation, because of the establishment of oxygen-depleted conditions [28]. Oxygen depletion achieved not only by the inhibition of photosystem II activity, but was also induced by the combinational transfer of electrons from reduced dcps to ubiquinone [28] through the respiratory mechanism of mitochondria (Figure 2C). The enhanced energy production from the respiration and the photosynthetic H_2_-production is invested mainly to dcps-biodegradation rather than to microalgal growth (Figure 6).

Using the [glc]-treatment, where most mono- and dichlorophenols can be biodegraded, an interesting bioenergetic biodegradation strategy appeared that was manipulated by the toxicity of the phenolic compound. Figure 5 presents a comparison between the catabolism of each of the dcps and the corresponding intermediates that produced during the biodegradation procedure. Considering the above results, it is clear that when the toxicity exceeds a certain threshold (such as dcps compared to cps), the microalgae decrease their biomass production (Figure 5A) in order to use the available energy for the biodegradation of the (degradable) dcps (Figure 5B). This means that the biodegradation of the toxic phenolic compounds gains absolute priority for the microalgae, whereas growth is of secondary concern under these circumstances. On the other hand, lower toxicities (cps) lead microalgae to use more energy for biomass increase and less in biodegradation (Figure 5). This is the main reason why all dcps present higher biodegradation levels per cell compared to the corresponding cps (Figure 5B) and also why the dcps of group II show higher biodegradation per packed cell volume than dcps from the lower toxic group I (Figure 5B). The above results are summarized in a simplified model in Figure 6 and in a comparative bioenergetics model between the group I and group II dcps biodegradation (Figure 7). The information for the model in Figure 7 is based on the findings of this contribution as well as on our recently published results [46].

The dcps of group I reduce the photosynthetic activity and the produced energy from photosynthesis and respiration (Figure 2) is invested mainly to growth (Figure 1), rather than to biodegradation (Figures 6 and 7A). The biodegradation of group II seems to be more complicated. Recently, we found that reduced dcps of group II, according to their redox potential, take place as electron donors to the photosynthetic electron flow, close to the plastoquinone pool (PQ). In parallel, they block the activity of photosystem II and the release of O_2_, leading to the establishment of oxygen-depleted conditions. Additionally, the first step of dcps-biodegradation is the dcps-reduction that supports a continuous circuit between oxidized and reduced dcps, which continuously promotes strong electron flow to PQ-pool, and in turn to ferredoxin (Fd). As a result, hydrogen production is induced strongly and continuously by the hydrogenase activation, because of the establishment of oxygen-depleted conditions [46] (Figure 7B). Oxygen depletion was not only achieved by the inhibition of photosystem II activity, but was also induced by the combinational transfer of electrons from reduced dcps to ubiquinone [46] through the respiratory mechanism of mitochondria (Figure 2C). The enhanced energy production from the respiration and the photosynthetic H_2_-production is invested mainly to dcps-biodegradation rather than to microalgal growth (Figures 6 and 7B).

It is known that dcp-biodegradation is a dynamic phenomenon. The intermediates of the biodegradation pathway change during the time and that possibly influence differently the biodegradation procedure. For this reason we tried to study all possible combinations of phenolic intermediates (like phenol, 2-cp and 4-cp, possible intermediates of 2,4-dcp biodegradation) in different treatments. The above results reveal a bioenergetic strategy (Table 2). Microalgae in limited energy reserves ([limit C]-treatment) start to biodegrade the most toxicant phenolic compound. In [CO_2_]-treatment where there are more energy reserves (compared to [limit C]-treatment) microalgae once again biodegrade the most toxicant phenolic compound only when in the culture medium there are intermediate metabolic compounds of the most toxicant one (cometabolism) (Table 2). In [glc]-treatment, where there are further energy reserves compared to the above treatments, all phenolic compounds can be biodegraded. The phenol treatment is an apparent exception, where microalgae prefer consuming glucose instead of phenol biodegradation as we have already explained in previous publication [45].

Another interesting observation is the cometabolic process of 2,4-dcp, 4-cp and phenol, in contrast to 2,4-dcp and 2-cp. The indication of Boyd and Shelton (1984) [59] regarding the bioconversion of 2,4-dcp to 4-cp, could be the main explanation of the above observation. Microalgae use energy to biodegrade phenolic compounds that follow the same metabolic pathway (2,4-dcp → 4-cp → phenol). Only if the energy reserves become plentiful, the microalgae consume energy for both metabolic pathways (Table 2– [glc]-treatment).

All the above show clearly that microalgae seem to be small and “smart” bioenergetic “machines”, that have the ability to continuously “calculate” the energy reserves and “use” the most energetically advantageous biodegradation strategy. We tried to manipulate the above fact, changing the energy reserves and as a result the chosen strategy, in order to take advantage of their abilities in detoxifying the environment or/and producing more valuable energy sources such as hydrogen.

## Materials and Methods

### Organism and Growth Conditions

Axenic cultures of the unicellular green alga *Scenedesmus obliquus*, wild type D3 [61] were autotrophically grown in liquid culture medium [62] and maintained for one week in controlled temperature (30°C) and light (150 µmol m^−2^ s^−1^) conditions. The cultures were continuously percolated with air for CO_2_ supply and sedimentation avoidance.

Axenic subcultures with an initial concentration of 1.5 µL packed cell volume (PCV) mL^−1^ were distributed into 100 mL hermitically sealed bottles (diameter 5 cm, height 9.5 cm) with septa for all the experimental procedures. The final culture volume in each bottle was 50 mL. The experiments were performed in a temperature-controlled room (30°C) at a light intensity of 50–60 µmol m^−2^ s^−1^.

Phenolic compounds (2,3-dichlorophenol; 2,4-dichlorophenol; 2,5-dichlorophenol; 2,6-dichlorophenol; 3,4-dichlorophenol and 3,5-dichlorophenol) were dissolved in methanol and added in concentrations of 0.15 mM. The corresponding methanol amount was also added in the control cultures. The above phenolic compounds concentrations were tested for their effects on algal cultures during the entire incubation time of 5 days in four different carbon sources. For the first treatment [limit C] a limited concentration of 0.036% CO_2_ (in air) was used. For the second treatment [CO_2_] 10% CO_2_ (in air) was used as inorganic carbon source [63]. For the third treatment [glc] a final concentration of 5 g/L glucose [62] was employed as organic carbon source, while for the last treatment [CO_2_+glc] a combination of both prementioned carbon sources was used.

Samples were collected daily in the same time in sterile conditions using sterile needles without opening the bottles, via the septum on the top of the bottle.

All the cultures were tested for possible contamination with bacteria and fungi before (mother cultures) and after the treatments (fifth incubation day) microscopically.

### Fluorescence Induction Measurements

The molecular structure and function of the photosynthetic apparatus was tested using fluorescence induction measurements by Handy Plant Efficiency Analyser, PEA (Hansatech Instruments, Kings’s Lynn, Norfolk, UK). This method is based on the measurement of a fast fluorescence transient with a 10 µs resolution in a time span of 40 µs to 1 s. Fluorescence was measured at 12-bit resolution and excited by three light-emitting diodes providing a saturated light intensity of 3000 µmol m^−2^ s^−1^ of red (650 nm) light. The JIP method of Strasser and Strasser (1995) [64] was used for the determination of the maximum yield of primary photochemistry (F_v_/F_m_), the functional antenna size per active reaction center (ABS/RC), the dissipation energy per active reaction center (DI_o_/RC) and the density of active photosynthetic reaction centers (RC/CS_o_).

### Measurements of Maximal Photosynthetic and Respiratory Rates

A Clark type electrode system (Hansatech Instruments, Kings’s Lynn, Norfolk, UK) was used for the determination of the maximal photosynthetic and respiratory rates, according to the method of Delieu and Walker (1981) [65]. The actinic light (500 µmol m^−2^ s^−1^) was generated with a light source (MILLE LUCE M1000) and a sensitive PAR/temperature sensor (Hansatech, Quantitherm) was used for the determination of its intensity. The infrared part of the applied irradiation was filtered off by inserting a 2% CuSO_4_-containing cuvette (4 cm path length) into the light beam. The cell suspension was adjusted before each measurement to 10 µL PCV mL^−1^.

### Quantitative Determination of Phenolic Compounds by HPLC

The isocratic HPLC-method of Lovell et al. (2002) [66] was used for the phenolic compounds analysis. Culture samples were centrifuged for 5 min at 1500 g and the supernatants injected into a Liquid Chromatography apparatus (Shimadzu LC-10AD) equipped with a diode array detector (Shimadzu SPD-M10A) and a narrow-bore column (C18, 2.1×150 mm, 5 µm particle size, hypersil, SUPELCO). The mobile phase was methanol : water : acetic acid (50∶49:1) at a flow rate of 0.2 mL min^−1^. The detection was carried out by measuring absorbance at 280 nm and the quantification by integration of known quantities of phenolic compounds.

### Growth Determination

The culture’s growth rate was estimated by measuring the packed cell volume (PCV) of the culture according to the method of Senger and Brinkmann (1986) [67]. The PCV of a cell suspension was determined by centrifugation at 1500 g for 5 min using haematocrite tubes and expressed as µL PCV (mL culture)^−1^.

### Data Analysis

Each treatment included three independent bottles and two samplings were carried out of each individual bottle. Standard deviations of the average values are presented on diagrams.
