# Correction: “Rational” Management of Dichlorophenols Biodegradation by the Microalga *Scenedesmus obliquus*

**DOI:** 10.1371/annotation/f16a8ba7-3989-4b58-aa7e-8d792e7d7ced

**Published:** 2013-11-04

**Authors:** Aikaterini Papazi, Kiriakos Kotzabasis

An error was introduced in the preparation of this article for publication. In the PDF version, on page 9, the third section of Table 2 reads [limit C]. The correct heading for the third section is [glc]. 

